# Genome-Wide Association of Bipolar Disorder Suggests an Enrichment of Replicable Associations in Regions near Genes

**DOI:** 10.1371/journal.pgen.1002134

**Published:** 2011-06-30

**Authors:** Erin N. Smith, Daniel L. Koller, Corrie Panganiban, Szabolcs Szelinger, Peng Zhang, Judith A. Badner, Thomas B. Barrett, Wade H. Berrettini, Cinnamon S. Bloss, William Byerley, William Coryell, Howard J. Edenberg, Tatiana Foroud, Elliot S. Gershon, Tiffany A. Greenwood, Yiran Guo, Maria Hipolito, Brendan J. Keating, William B. Lawson, Chunyu Liu, Pamela B. Mahon, Melvin G. McInnis, Francis J. McMahon, Rebecca McKinney, Sarah S. Murray, Caroline M. Nievergelt, John I. Nurnberger, Evaristus A. Nwulia, James B. Potash, John Rice, Thomas G. Schulze, William A. Scheftner, Paul D. Shilling, Peter P. Zandi, Sebastian Zöllner, David W. Craig, Nicholas J. Schork, John R. Kelsoe

**Affiliations:** 1 Scripps Genomic Medicine and Scripps Translational Science Institute, La Jolla, California, United States of America; 2 Department of Pediatrics and Rady's Children's Hospital, School of Medicine, University of California San Diego, La Jolla, California, United States of America; 3 Scripps Health, La Jolla, California, United States of America; 4 Department of Medical and Molecular Genetics, Indiana University School of Medicine, Indianapolis, Indiana, United States of America; 5 Neurogenomics Division, The Translational Genomics Research Institute, Phoenix, Arizona, United States of America; 6 Department of Psychiatry, University of Michigan, Ann Arbor, Michigan, United States of America; 7 Department of Psychiatry, University of Chicago, Chicago, Illinois, United States of America; 8 Department of Psychiatry, Portland VA Medical Center, Portland, Oregon, United States of America; 9 Department of Psychiatry, University of Pennsylvania, Philadelphia, Pennsylvania, United States of America; 10 Department of Psychiatry, University of California San Francisco, San Francisco, California, United States of America; 11 Department of Psychiatry, University of Iowa, Iowa City, Iowa, United States of America; 12 Department of Biochemistry and Molecular Biology, Indiana University School of Medicine, Indianapolis, Indiana, United States of America; 13 Department of Psychiatry, University of California San Diego, La Jolla, California, United States of America; 14 Center for Applied Genomics, Abramson Research Center, The Children's Hospital of Philadelphia, Philadelphia, Pennsylvania, United States of America; 15 Beijing Genomics Institute at Shenzhen, Shenzhen, China; 16 Department of Psychiatry, Howard University, Washington, D.C., United States of America; 17 Cardiovascular Institute, University of Pennsylvania School of Medicine, Philadelphia, Pennsylvania, United States of America; 18 The Institute for Translational Medicine and Therapeutics, School of Medicine, University of Pennsylvania, Philadelphia, Pennsylvania, United States of America; 19 Department of Psychiatry, Johns Hopkins School of Medicine, Baltimore, Maryland, United States of America; 20 Mood and Anxiety Section, Human Genetics Branch, National Institute of Mental Health Intramural Research Program, National Institutes of Health, United States Department of Health and Human Services, Bethesda, Maryland, United States of America; 21 Department of Psychiatry, Indiana University School of Medicine, Indianapolis, Indiana, United States of America; 22 Division of Biostatistics, Washington University, St. Louis, Missouri, United States of America; 23 Department of Psychiatry and Psychotherapy, Georg-August-University Göttingen, Göttingen, Germany; 24 Department of Psychiatry, Rush University, Chicago, Illinois, United States of America; 25 Department of Molecular and Experimental Medicine, The Scripps Research Institute, La Jolla, California, United States of America; 26 Department of Psychiatry, VA San Diego Healthcare System, La Jolla, California, United States of America; Georgia Institute of Technology, United States of America

## Abstract

Although a highly heritable and disabling disease, bipolar disorder's (BD) genetic variants have been challenging to identify. We present new genotype data for 1,190 cases and 401 controls and perform a genome-wide association study including additional samples for a total of 2,191 cases and 1,434 controls. We do not detect genome-wide significant associations for individual loci; however, across all SNPs, we show an association between the power to detect effects calculated from a previous genome-wide association study and evidence for replication (P = 1.5×10^−7^). To demonstrate that this result is not likely to be a false positive, we analyze replication rates in a large meta-analysis of height and show that, in a large enough study, associations replicate as a function of power, approaching a linear relationship. Within BD, SNPs near exons exhibit a greater probability of replication, supporting an enrichment of reproducible associations near functional regions of genes. These results indicate that there is likely common genetic variation associated with BD near exons (±10 kb) that could be identified in larger studies and, further, provide a framework for assessing the potential for replication when combining results from multiple studies.

## Author Summary

Bipolar disorder (BD) is a highly heritable disease that has been difficult to characterize genetically. We have genotyped 1,190 BD cases and 401 controls to find regions of the genome associated with BD. After combining these data with previously existing genotyped samples, we did not find any genome-wide significant associations. However, when we used an additional study to prioritize loci for replication and meta-analysis purposes, we found that we were more likely to see an association in our sample with variants for which we had the highest power. We quantified this effect using logistic regression and saw a strong association between power to detect an effect based on an initial study's results and replication P-value in a second study (P = 1.5×10^−7^), supporting the presence of shared genetic risk factors across the studies. Moreover, this association was stronger when we restricted analysis to SNPs near coding regions, and it was further enriched when SNPs had the same direction of effect in both studies. This result supports the presence of genetic factors underlying BD near exons whose collective effect results in a detectable signal and provides a framework for assessing the potential for replication when combining results from multiple studies.

## Introduction

Genome-wide association studies (GWAS) have been responsible for the elucidation of hundreds of loci associated with common human diseases, in some cases aiding in the prediction of individual disease susceptibility, but primarily allowing for a better biological understanding of disease [1], [2]. Although effect sizes of associated variants identified in these studies have been small to modest, it has been suggested that many more loci of even smaller effect may be detected with larger datasets [3] based on the distribution of associated variant frequencies and effect sizes and, in the case of height, these non-significant effects can add up to a large proportion of the variance explained [4]. This has been borne out with recent GWAS using hundreds of thousands of individuals [5]–[7]. A challenge is determining whether additional samples are worth genotyping for common variation when initial results with modest sample sizes do not result in genome-wide significant effects. Here, we analyze effects across multiple GWAS with sub-significant P-values to determine whether there is a true underlying genetic signal tagged by common variation present across studies. Since true effects will tend to replicate across studies as a function of power, we can test the hypothesis that there is an underlying genetic signal for a trait by testing whether replication of an association with a variant across studies is a function of the power to detect that variant based on that variant's frequency and effect size as determined from a single study. It has been suggested as an alternative to a Bonferroni-based approach to genome-wide significance that P-values be interpreted in the context of power [8]. We assume that the association statistics in a previous study provide prior information about the potential for replicability of associations, by estimating the power to detect SNP effects from the frequency and effect sizes determined in an initial data set on a SNP-by-SNP basis and then testing associations with those SNPs in a test data set, and can thereby focus on the variation that is most likely to be truly associated.

Bipolar disorder (BD) is a major psychiatric disorder affecting approximately 1% of the population. Patients with BD suffer extreme mood swings between mania and depression, and 17% suicide. BD is highly heritable, but has not easily yielded genetic loci from family and population based mapping strategies [9], [10]. Multiple genome-wide association studies [11]–[14] have highlighted compelling candidates for BD without reaching genome-wide significance. *ANK3* and *CACNAC1* were identified at genome-wide significance through the combination of multiple GWAS [15], and a meta-analysis identified a region at 3p21.1 associated with a combined sample of individuals with BD or major depressive disorder [16]. Intriguing, however, are results that suggest a shared polygenic basis with schizophrenia, with effects over many loci, each contributing a small effect [17]. By considering the development and application of a multilocus schizophrenia-based genetic risk ‘score’ across many SNPs – many that were not significant in a single locus analysis – Purcell et al. [17] were able to predict BD case-control status with a non-zero probability, indicating a probable polygenic basis for Schizophrenia. In this work, we assess the relationship between the power to detect a SNP based on association statistics observed in one study and replication in another study. We assess this relationship in a way that allows for the assessment of collections of SNPs in defined genomic regions in order to test hypotheses about the nature of genetic variation mediating BD susceptibility.

We apply this strategy to new BD GWAS data to show that it is possible to identify replicable genetic signals in circumscribed regions of the genome that would not be captured by single locus analyses in a single GWAS data set at genome-wide significance levels. Essentially, we assess the consistency of effects at different loci across studies by calculating the power to detect an effect in a “discovery” study and comparing the results to observed associations in a “test” study. Power encompasses both allele frequency and penetrance or effect size, and therefore is a single measure of the likelihood of replication. If there is a true underlying signal common to the two datasets that is tagged by common variation interrogated in the genotyping chips used in the studies, then one would expect to see greater evidence for replication at loci for which there is greater power to detect an effect. If there were no true underlying signal, one would expect to see no association between power calculated from an initial study's findings and replication in an independent data set. Because we characterize trends across many SNPs without identifying individually significant effects, this approach has similarities to the false discovery rate [18] approach, but uses an unrelated study to prioritize markers.

## Results

We first considered the results of an association study involving 1,190 newly genotyped BD cases from the Bipolar Genome Study (BiGS) and 401 controls, referred to as the ‘TGEN’ sample (Table S1). These samples were collected through the same mechanisms as 1,001 cases and 1,033 controls of European Ancestry genotyped through the GAIN initiative [12]. However, while most of the samples in GAIN were collected as part of extended families or sib-pairs, the TGEN samples were primarily selected without regard to family history. We combined the GAIN and TGEN samples, for a total of 2,191 BD cases and 1,434 controls. We performed GWAS (Figure S1) and report the top regions at P<10^−5^ (Table S2 and Figure S2). Although none of the associations reached genome-wide significance of 5×10^−8^, we note that 1 SNP in the region near the voltage-dependent calcium channel gene *CACNA2D1* was associated at P = 5.9×10^−6^ (rs2367911). A related gene, *CACNA1C*, reached genome-wide significance in a large meta-analysis [15], but was not significant in this study (Table S3). We assessed replication of loci implicated in other GWAS and show consistent support with the imputed SNP rs10994336 at *ANK3* (P = 0.02), as well as the genotyped SNP rs9804190 (P = 0.02) that has been suggested to signal an alternate allele [14] (Table S3). We performed a fixed effects meta-analysis with SNPs that overlapped in the Wellcome Trust Case Control Consortium (WTCCC) [8] BD dataset (Figure S1 and Table S4). There were no genome-wide significant associations, with the strongest association at chromosome 2 (peak SNP rs12618769, P = 1.0×10^−6^). Although many of the top associations changed, there was an overall high correlation (r = 0.42) between −log P-values across the GAIN-TGEN and GAIN-TGEN-WTCCC meta-analyses. We scored individuals in the GAIN+TGEN study based on the observed odds ratios in WTCCC across all markers to test for polygenic effects [17]. We saw a significant association when all SNPs, SNPs pruned for linkage equilibrium (r^2^<0.5), or SNPs pruned for independent associations using PLINK's “clump” procedure were used (All: P = 2.3×10^−20^, LE: P = 1.7×10^−17^, clumped: P = 5.9×10^−18^), with all SNPs explaining 3.3% of the variation in diagnosis (Figure S3).

Because the multilocus scoring method suggested an underlying polygenic influence on BD, that did not include SNPs that were individually significantly associated with BD at genome-wide significance levels, we hypothesized that we were underpowered to detect single locus effects given our sample size. If, however, effect sizes for the SNPs have been well estimated in the GWAS considered, then we would expect that we would observe associations in one data set as a function of power to detect effects based on information obtained in a different data set; i.e., variants that we have 80% power to detect based on one data set will be observed to be associated 80% of the time in other data sets. We thus assessed the power to detect SNP effects obtained in one GWAS data set and applied this information to others (Figure S4). To do this, we estimated effect sizes based on a discovery GWAS data set, calculated power to detect those effects, and tested whether SNPs with the greatest power exhibited replicable associations in a second GWAS dataset.

**Figure 1:**
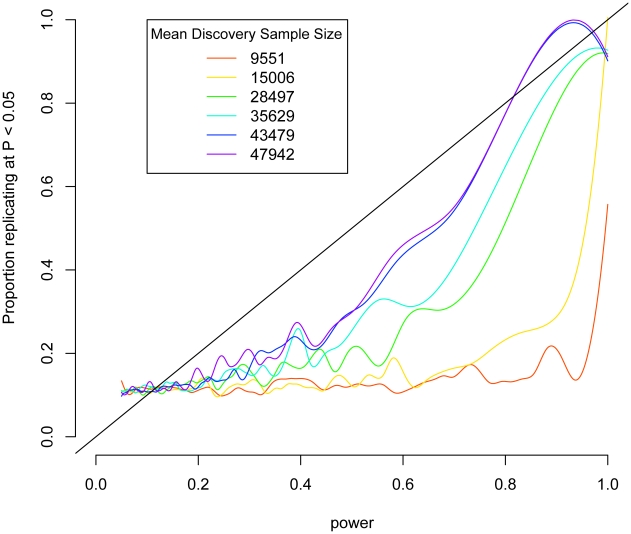
Replication as a function of power in height meta-analysis. Power to detect associations in test data sets was calculated based on observed effects in discovery subsets of the IBC height collection and is plotted against association at P<0.05 in the test data. Lines are smoothed splines indicating the proportion of SNPs that replicate at P<0.05 across varying power. Smaller subsets of the discovery data set are shown in rainbow colors.

As a proof of principle of this strategy, we considered data from a recently published meta-analysis on height [19]. Up to 53,394 individuals were genotyped on a cardiovascular disease-focused array [20], which contains 49,320 SNPs. These results were followed up in 37,052 additional samples genotyped on the same array. For each SNP, we calculated power to detect the effect in the test sample based on the effect, allele frequency, and standard deviation of height in the discovery sample. We show a strong association with almost a linear relationship between power and replication (Figure 1). If the discovery data set is restricted to fewer people, worsening the estimate of the effect size, the association decays (Figure 1). These results suggest that if the discovery sample is large enough to give a good estimate of the effect size, then we should expect replication to show a linear relationship with power. We verified this by simulating hypothetical data based on the effect sizes observed a sample of SNPs chosen from the BD and height data sets and show that if the observed effects were real, that we would expect to see replication rates linearly associated with power approximating a slope of 1 (Figure S5).

**Figure 2:**
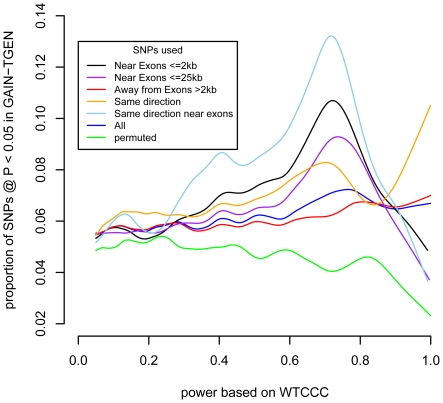
SNPs near exons show a stronger enrichment at P<0.05 as a function of power. For different classes of SNPs, the smoothed spline is shown for the proportion of SNPs showing association at P<0.05 in the GAIN+TGEN dataset as a function of power based on the WTCCC dataset.

We applied this test to our BD samples. Of the SNPs that were shared between the WTCCC and GAIN-TGEN, we had 60% or higher power at an alpha of 0.05 to detect associations of the same or larger effect at only 7,277/364,259 (2.0%) SNPs, if the WTCCC effect sizes were true. We tested the hypothesis that the probability of association at P<0.05 in GAIN+TGEN was associated with the power to detect them based on the WTCCC data using logistic regression. We found that we were more likely to replicate associations at P<0.05 when we had more power to detect them (Figure 2, blue line; logistic P = 1.5×10^−7^). This P-value does not require multiple-testing correction since we are not analyzing SNPs individually, but are rather testing a single hypothesis: the correlation between power based on one study and association strength in another. This trend was not observed when case/control status was permuted in the GAIN+TGEN combined sample (Figure 2, green line; P = 0.32). When we restricted our analysis to a subset of SNPs that are in linkage equilibrium (r^2^<0.5), we still see a significant association (P = 0.01). These results are consistent with the notion that multiple variants, each likely of small effect, contribute to BD. It might also be the case that the genotyped loci are tagging multiple rare variants that contribute to a polygenic effect [21]. Excluding regions covering *ANK3*, *CACNA1C*, and 15q14 (+/−1 Mb) that have been highlighted in a meta-analysis of BD GWAS [15] did not attenuate the association (logistic P = 1.4×10^−7^). This indicates that there is an underlying genetic signal for BD shared between the WTCCC and GAIN+TGEN studies within yet-to-be-described regions that are tagged by common variation.

We estimated the number of SNPs driving this effect by counting the excess SNPs in the highest power deciles (Figure S6). Among SNPs for which we have 60% power or higher, we observe 503/7,277 (6.9%) associated at P<0.05. This is an excess of 89 SNPs more than the 414 that we would expect from the average rate of replication across all SNPs, 5.69%. Since the majority (414/503, 82%) of the SNPs are likely to be false, however, we cannot specifically identify which SNPs are contributing to the excess association. Additionally, since some of these SNPs are not independent, we cannot say how many loci might be causally associated with BD.

Recent advances in sequencing and exon targeting have made exon sequencing more feasible. We tested whether there was an excess of replicating SNPs near coding regions. We stratified the SNPs by their location relative to exons (within 2 kb; this amounted to 15% of all SNPs). We then tested whether SNPs near exons showed stronger associations between discovery set-based power to replicate and test set replication (Figure 2, black line). At the higher levels of discovery-based power, a higher proportion of SNPs near exons yielded P<0.05 than did SNPs that are further away from exons (Figure 2, red line). We quantified this difference using logistic regression, testing the interaction between power and whether a SNP was close to an exon (logistic regression SNP location×power interaction, P = 7.8×10^−6^). Extending the distance between SNP and exon to 25 kb attenuated the enrichment at higher power levels (Figure 2, purple line), but the interaction term remained significant (P = 8.4×10^−5^). When we investigated a range of distances, the enrichment was strongest up to 10 kb, but SNPs within exons were not enriched for replication (Figure S7). This is consistent with variation in nearby sequences, possibly regulatory in nature, being associated with the disease. This suggests that the regions near exons are likely to be enriched for variants associated with BD. This does not imply that disease-associated variation is exonic, but that the portion of the genome that is near genes also contains proportionally more variation likely to be associated with disease.

As a negative control, we used power derived from phenotypes in the WTCCC other than BD and performed the same tests (Table 1). These other phenotypes did not show the same level of reproducibility as BD, although some P-values were observed at P<0.05/7, these were weak compared to the BD-derived values. This suggests that the enrichment of associated SNPs is specific to BD. SNPs were more likely replicate at P<0.05 if the effects seen in GAIN+TGEN were in the same direction as those seen in WTCCC (P = 1.3×10^−21^, Table S5). This effect was also dependent on power, consistent with true associations having the same direction of effect (P = 1.9×10^−6^). Using other WTCCC phenotypes as input, we usually saw no association between the consistency of direction of effect and replication in GAIN+TGEN (Table S5). We did see an association with Crohn's Disease (P = 3.0×10^−8^), but this effect was independent of power (interaction P = 0.65). These less strongly associated relationships could be explained by subtle underlying shared genetics, but may also be artifactual as they were not consistent with patterns observed with overall genotype correlations showing similarity between BD, CAD, and T2D [22].

**Table 1:** P-values for association between power based on WTCCC GWAS and replication in the GAIN+TGEN study is significant for BD and dependent on the location of the SNPs relative to exons.

Model	Predictor	SNP subset	BD	CD	CAD	HT	RA	T1D	T2D
I	power	All	1.5×10^−7^	0.051	0.621	0.605	0.009	0.101	0.003
II	power	All	0.003	0.059	0.048	0.743	0.026	0.192	0.096
II	near exon	All	0.008	0.534	0.017	0.867	0.876	0.894	0.140
II	powerXnear exon	All	1.7×10^−5^	0.785	1.1×10^−4^	0.722	0.710	0.681	0.009
III	power	Same Direction	7.0×10^−13^	0.080	0.347	0.195	0.692	0.896	0.306
IV	power	Same Direction	9.2×10^−6^	0.187	0.048	0.076	0.265	0.469	0.366
IV	near exon	Same Direction	7.0×10^−4^	0.799	0.261	0.607	0.578	0.526	0.270
IV	powerXnear exon	Same Direction	8.7×10−^7^	0.523	0.005	0.159	0.068	0.159	0.957

Header abbreviations: BD: bipolar disorder, CD: Crohn's disease, CAD: coronary artery disease, HT: hypertension, RA: rheumatoid arthritis, T1D: type 1 diabetes, T2D: type 2 diabetes.

While the use of P-value<0.05 represents a moderately stringent cutoff, showing effects in the same direction is a less stringent criterion. If we restrict our analyses to only those SNPs where effects were in the same direction in both studies, we see even stronger associations with BD and no association with the other phenotypes (Table 1). The association with power is similarly enriched (Figure 2). This adds support for the observation that these effects are consistent across studies that are likely to reflect underlying true variation associated with BD.

SNPs with very high power (>90%) based on WTCCC BD effects were less likely to replicate than SNPs at powers between 80–90%. In addition to noise due to smaller numbers of SNPs at the higher level, there may be artifactual associations in the strongest associations of the WTCCC: many of the top associated SNPs in the original study required filtering, but cluster plots were not inspected for those associations that were not at P<10^−7^ [8]. We switched the sample used to calculate power, using GAIN+TGEN as the basis for the OR and MAF, and calculated association in the WTCCC sample, and did not see this effect (Figure S8). The overall association is significant, albeit weaker (P_power_ = 0.002, P_location×power interaction_ = 2.0×10^−7^).

## Discussion

We report a complementary approach to standard meta-analysis when there is an existing, unrelated study that can be leveraged to assess the consistency of effects across studies. This analysis does not specify which SNPs are associated, but investigates trends among the SNPs and their association strengths. By analyzing results in the context of what one study suggests is the power to detect effects in another study, association signals likely to be of functional significance can be better partitioned.

Using an analysis of height GWAS data as a proof-of-principle, we showed that with enough samples in the discovery data set there was an almost linear relationship between replication P-values and power based on variant and effect size information obtained in a separate sample with a slope of 1. However, we did not see as strong of an effect with BD. This could be due to the small sample size in WTCCC-BD, relative to the height data we had at our disposal; the association may increase with sample size.

In this analysis, we assume that the same variation that is likely to be causal in one study is likely to be causal in the other, and that both studies have similar linkage disequilibrium structure. Population-specific variation or variation that has population-dependent effects would not be expected to replicate to the same extent. Phenotypic heterogeneity across populations, which may be more of a challenge for psychiatric diseases than it is for height, will also contribute to low levels of replication. In the case of heterogeneity, one would expect that the relationship between replication and power would not approach a slope of 1 as sample size increases.

Although we use common variation to tag these associations, the underlying functional variation may be rare or common, as collections of rare variants of stronger effect can produce an association signal consistent with observed effect sizes [21]. Further studies that include deep sequencing would be required to identify these variants. As applied to BD, this analysis supports the presence of replicable and potential functional variation associated with BD that is enriched in regions near genes. However, the enrichment signal was not present within exons but rather was observed only when regions up to 10 kb around exons were included in the analysis. This suggests that sequencing of individuals with BD should include noncoding regions near genes.

The model of polygenic inheritance suggests that there are many loci throughout the genome, each with small effect, that influence phenotypic expression. Our result for BD does not rule out the model of many loci, but suggests that for this disease, truly associated variation may be enriched near genes. Thus, the genetic architecture of BD that is tagged by common variation does not appear to be evenly distributed throughout the genome, but may reside in circumscribed regions.

Many recent studies have been pursued to better understand if non-genome-wide significant variation can still be considered to harbor phenotypically-relevant information. For example, Yang *et al.* [4] quantified variation explained by all SNPs by fitting a single regression model that included thousands of variants in order to assess the collective effect of these variants on height and estimated the variation in height explained by these variants. Park *et al.* [3] took a different approach and used the distribution of effect sizes at genome-wide significant loci in conjunction with the power to detect those effects to extrapolate the distribution of undetected genetic loci. Our approach differs from these methods in a number of ways. First, we do not require individual level data and only require summary level results, which can obviate the need for individual data use restrictions. Second, we do not require genome-wide significant results to estimate a true effect size distribution, which is helpful when there are few to no significant associations. Third, because the replication vs. power relationship approaches a slope of 1 when the effect sizes are real and reproducible, this approach provides and alternative framework for understanding the extent of the reliability of signals even when they are not genome-wide significant.

Given the number of inconclusive GWAS with marginal results in need of interpretation, we feel that the approach described in this paper provides an important tool for assessing whether there is an underlying genetic basis for a phenotype and/or whether additional samples might be needed to detect genetic associations. Studies that are too underpowered to detect any replication signal, however, may require additional samples before applying this approach. Additionally, in the context of sequence data, specific groups of variants can be tested for stronger associations between power and replication. The method is easy to apply in the context of a meta-analysis where association results are present for large numbers of SNPs.

## Materials and Methods

### Ethics Statement

All eleven collection sites in the BiGS Consortium received IRB approval for subject ascertainment, assessment, and collection of DNA for genetic studies. All participating subjects signed a statement of informed consent.

### Study Subjects

The subjects used in the GAIN [12] and WTCCC [8] samples have been previously described. The TGEN cases consisted of unrelated individuals from the “Wave 5” collection of the Bipolar Consortium, which included 1,310 unrelated DNA samples from families ascertained through probands with DSM IV-defined BPI disorder [12], 1,190 of which ultimately passed QC measures. While GAIN samples were primarily from larger families with multiple BD cases or sib-pairs, TGEN samples were primarily population-based and were not required to have a family history. Controls were collected by NorthShore University HealthSystem, Evanston, IL, R01 MH59571, Pablo V. Gejman, M.D. (Collaboration Coordinator; PI) as part of a collaborative R01 application comprised of ten sites (see Acknowledgements).

### Genome-Wide SNP Genotyping

Genomic DNA samples were analyzed on the Genome-Wide Human SNP 6.0 Array (Affymetrix, Inc. Santa Clara, CA) according to the manufacturer's protocols (Affymetrix Genome-Wide Human SNP Nsp/Sty 6.0 User Guide;Rev. 1 2007). Before the initiation of the assay, 50 ng of genomic DNA from each sample was examined qualitatively on a 1% Tris-acetate-EDTA agarose gel for visual signs of degradation. Any degraded DNA samples were excluded from further analysis (∼3%). Samples were quantitated by Spectrometry and diluted to 50 ng/µl in reduced EDTA TE buffer (10 mM Tris HCL, 0.1 mM EDTA, pH 8.0). 250 ng of DNA was then aliquotted into two 96-well reaction plates and digested with either Sty or Nsp restriction enzymes (New England Biolabs, Inc. Ipswich, MA) for 2 hours at 37°C followed by 65°C for 20 min. Sty and Nsp digested samples were then ligated to either the Sty 1 or the Nsp 1 adaptor (Affymetrix), respectively, with T4 DNA Ligase (New England Biolabs) for 3 hours at 16°C then 20 min at 70°C. The ligated samples were then diluted in molecular-grade water and subaliquotted into 3 (Sty) or 4 (Nsp) 96 well PCR plates. PCR was performed using PCR Primer 002 (Affymetrix) and Titanium Taq DNA Polymerase (Clontech, Mountain View, CA) with the following thermal cycling parameters: 1. 94°C for 3 min., 2. 30 cycles of 94°C for 30 sec., 60°C for 30 sec., and 68°C for 15 sec., and 3. 68°C for 7 min. Like samples for all Sty and Nsp reactions were pooled into a single deep well plate, the DNA was bound to Agencourt AMPure beads (Beckman Coulter, Inc. Berea, CA), placed into MultiScreen filter plates (Millipore, Billerica, MA), washed with 75% ethanol and eluted with Buffer EB (QIAGEN, Valencia, CA). Purified samples were then fragmented using Fragmentation Reagent (Affymetrix) and incubated at 37°C for 35 min. then at 95°C for 15 min. Fragmented samples were labeled with DNA Labeling Reagent (Affymetrix) and TdT Enzyme (New England Biolabs) at 37°C for 4 hours followed by 95°C for 15 min. The samples were denatured at 95°C for 10 min. and held at 49°C until they were loaded on to the arrays. The arrays were placed into the hybridization oven at 50°C and 60 rpm for 16 to 18 hours. Arrays were then washed, stained and immediately imaged on the GeneChip Scanner 3000 (Affymetrix). Birdsuite was used to call SNP genotypes from CEL files. Initial quality control measures consisted of gender-checks and a custom SNP fingerprinting approach to identify potentially duplicated or related individuals.

### Genotyping Quality Control

1,858 samples [1,310 Bipolar cases, 408 controls,140 technical controls (42 case replicates, 43 control replicates, 19 HapMap individuals, and 36 parents from 19 case families)] passed a call rate threshold of 97%, QC contrast of 0.40, and gender consistency. We further removed samples that did not have a diagnosis of Bipolar Disorder I or Schizoaffective Bipolar Disorder (78 cases filtered), that were outliers on the first 2 coordinates of an MDS plot including HapMap 3 individuals (N = 6 controls and 35 cases), that showed poor concordance between duplicates (3 individuals out of 85 pairs), that appeared to be more similar to another individual in the GAIN study than expected (PI_HAT>0.15) (6 cases that were related to someone in GAIN or appeared to be the same person that had entered the study twice), or that had high heterozygosity (>0.285 averaged across all markers, N = 1 case). A total of 1,190 cases and 401 controls remained and are included in the analysis.

SNPs were filtered for a lack of positional information from Affymetrix (N = 1,233), low minor allele frequency (<1%, N = 145,345), significant deviation from Hardy-Weinberg equilibrium in controls (P<10^−6^, 592), low call rate (<95%, N = 34,930), poor duplicate concordance (>2 heterozygote or homozygote errors, 16,541), or >1 Mendelian error within families (N = 1,348). A total of 178,413 SNPs were removed, leaving a final count of 728,187 SNPs. Of these, 703,019 also passed QC in GAIN and were included in the merged analysis. Genotypes are reported in genome forward orientation based on NCBI build 36 *via* the Affymetrix annotation file GenomeWideSNP_6.na27.annot.csv.

### Imputation

Genotype data was further filtered (MAF> = 5% and Hardy-Weinberg P<10^−6^ using all samples) and imputed to the CEU HapMap 2 (CEU_r22_b36_fwd) genotypes using MACH [23] (http://www.sph.umich.edu/csg/abecasis/MACH/index.html). Imputation results were filtered such that r^2^> = 0.30.

### Association

Association analysis was performed on the genotype data in PLINK [24] using logistic regression, adjusting for study in the GAIN+TGEN sample, and using the –dosage command with the predictor being the maximum likelihood estimate of the number of alleles at the locus (format = 1). Adjusting for up to 10 MDS components did not alter the genomic inflation factor, so they were not included as covariates.

### WTCCC Data

WTCCC genotype data was downloaded in TPED format and filtered as described in the accompanying documentation. Genotype calls were filtered based on CHIAMO quality scores (>0.90); SNPs were filtered according to SNP lists provided by the WTCCC, including SNPs that were excluded based on poor genotype clustering; and individuals were filtered according to individual lists provided by the WTCCC for a total of 459,075 SNPs, 1,868 bipolar disorder (BD) cases, 1,926 coronary artery disease (CAD) cases, 1,748 Crohn's disease (CD) cases, 1,952 hypertension (HT) cases, 1,860 rheumatoid arthritis (RA) cases, 1,963 type 1 diabetes (T1D) cases, 1,924 type 2 diabetes (T2D) cases, 1,458 United Kingdom Blood Services (NBS) controls, and 1,480 1958 British Birth Cohort (58C) controls.

### Meta-Analysis

Association for WTCCC samples was performed in PLINK using logistic regression without any covariates. For each phenotype, cases were analyzed against both (NBS and 58C) control sets. Meta-analysis was performed in PLINK using the –meta-analysis command. Fixed effects P-values are reported.

### Polygenic Scoring

SNPs that were genotyped in both WTCCC-BD and GAIN+TGEN were used to generate scores for each individual in GAIN+TGEN. Odds ratios from WTCCC-BD were natural log transformed and used as a score in the SNP scoring routine of PLINK. Subsets of SNPs achieving different P-value cutoffs were used such that weakly associated SNPs were progressively added to strongly associated SNPs. SNPs were pruned to linkage equilibrium (r^2^<0.5) using the “—indep-pairwise” command in PLINK with a sliding window of 50 SNPs and a 5 SNP step. SNPs were clumped to independent associations using the “—clump” command in PLINK. Index SNPs were selected at P<0.1, with a secondary threshold of P<0.1, r^2^<0.5, and a 250 kb window. Logistic regression was used to test for association between score and case-control status in R [25] (glm command). The lrm command was used to calculate a pseudo-R^2^ statistic.

### Height Meta-Analysis and Power Calculations

Summary-level data was obtained from a meta-analysis of height using a genotyping chip targeting genes related to cardiovascular disease, covering 49,320 SNPs and about 2,000 genes. We used the natural division of the Phase I cohort consisting of 53,394 individuals of European Ancestry as our discovery set and the collection of Phase II cohorts consisting of 37,052 individuals of European Ancestry as our test dataset. We performed meta-analysis on the Phase I and Phase II samples using METAL as described [19].

Power was calculated using the pwr.f2.test function from the pwr package in R with 1 degree of freedom in the numerator; N−2 degrees of freedom in the denominator, where N is the number of individuals with genotype data for the SNP in the test dataset; alpha of 0.05; and effect size f^2^. The effect size f^2^ was calculated as:
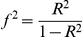
R^2^ was calculated as:
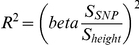
where beta is the effect size from the meta-analysis and s is standard deviation. In the study, height was expressed in cm and was not standardized to a z-score. The standard deviation of height was calculated as the sample size-weighted standard deviation across all discovery data sets (9.2 cm), and the standard deviation of the SNP was calculated from the allele frequencies assuming Hardy-Weinberg equilibrium. Because a proportion of SNPs were only tested in a small number of samples, we filtered the 20% of SNPs with the lowest sample sizes. To test the role of sample size on initial effect estimates, studies were progressively added to the discovery data and the meta-analysis was repeated on each subset of the data.

### BD Power Calculation

Power was calculated based on association results from WTCCC Bipolar cases and controls (NBS+58C) [26]. SNPs were additionally filtered for MAF (>1%). In order to calculate power, the non-centrality parameter was calculated given the odds ratio (OR) from WTCCC, minor allele frequency in WTCCC controls, and the number of case and control samples in the TGEN+GAIN combined sample [27]. OR and MAF were rounded to 2 decimals. Power was calculated using G*Power 3 [28] given the non-centrality parameter and an alpha of 0.05.

Associations between power and replication at P<0.05 or consistency of effect size were performed using logistic regression in R using the glm command (family = binomial(“logit”)). For plots, smoothing was performed using the smooth.spline function in R.

### Power Simulations

For both height and BD samples, we performed simulations in R to show that given the observed effect size, we would expect to see replication rates associated with power linearly with a slope of 1. We sampled 361 SNPs from each study across power levels, with 19 SNPs from each 5% power bracket. We simulated a population of 1,000,000 individuals with genotypes based on the allele frequency given Hardy-Weinberg expectations. For the BD case-control study, an individual's risk of disease was 1% multiplied by the odds ratio raised to the power of the number of risk alleles they carried. If this risk was greater than a random number between 0 and 1, then they were considered affected. For each SNP, we performed 100 permutations, sampling cases and controls in numbers to match the GAIN-TGEN sample and performed logistic regression. The observed replication rate is the proportion of tests that reached P<0.05. For the quantitative height example, a individual's baseline height in standard deviations was modeled using a random number as a quantile of the normal distribution using the command qnorm in R. The observed effect of the SNP was then multiplied by the number of risk alleles and added to the baseline height. For 100 permutations per SNP, a random sample of individuals corresponding to the number of individuals tested for that SNP in the Phase II study was taken and association was tested using linear regression.

### Enrichment near Exons

Exon location in the RefSeq genes was determined from refGene table (http://genome.ucsc.edu/cgi-bin/hgTables). For each SNP, the closest exon within 1 Mb was determined and the distance to that exon calculated. If a SNP was within an exon, a distance of 0 was used. An indicator variable of whether the SNP was within 2 kb or 25 kb of an exon was used in logistic regression.

## Supporting Information

Figure S1 Manhattan plots of GAIN+TGEN and Meta-analysis −logP values. Manhattan plots for A) GAIN+TGEN and B) GAIN+TGEN+WTCCC BP meta-analysis. Points are indicated as circles for genotyped data and triangles for imputed data. Points are larger and circled in pink if the P-value<10^−5^, also indicated by a pink dotted line.
(PDF)

Figure S2 Regional plots for GAIN+TGEN GWAS. Associations reaching P<10^−5^ in the GAIN+TGEN GWAS are shown with nearby markers. The X-axis indicates the chromosomal position based on NCBI build 36. Genes are shown based on their locations in RefSeq. Arrows indicate the direction of transcription. Points are color coded according to LD within the GAIN+TGEN study with the most associated SNP. For imputed markers, LD was calculated as correlation in PLINK using best estimates for genotypes. Recombination rate is indicated on the second y-axis and color-coded in light blue. Cluster plots for singleton SNPs such as rs17498753 and rs293969 were inspected and found to be of good quality.
(PDF)

Figure S3 Variance in diagnosis explained in GAIN+TGEN by score summed over using subsets of SNPs from the WTCCC-BD study. A score was calculated for each individual based on their genotype and the odds ratio of each SNP from the WTCCC-BD study. The score was used to predict case-control status in GAIN+TGEN and shown are the pseudo-R^2^ values from logistic regression for subsets of SNPs used to calculate a score. SNPs were grouped by P-value, with each category adding progressively more SNPs with weaker P-values until all SNPs are included. LD-pruned SNPs were pruned to be in linkage equilibrium (r^2^<0.5). Clumped SNPs were pruned to index SNPs to ensure independent associations.
(PDF)

Figure S4 Schematic of test for replication as a function of power.
(PDF)

Figure S5 Simulation studies show a linear relationship between power and replication. A sample of SNPs were selected from A) BD or B) height and the effects were simulated in a population of 1,000,000 individuals assuming a baseline prevalence of 1% for BD and a normal distribution for height. Random case-control (BD) or population-based (height) samples were taken such that they matched the observed sample sizes. For each SNP, 100 of these samples were taken and the observed replication rate corresponds to the proportion of samples where the P-value was less than 0.05. A line is drawn at y = x.
(PDF)

Figure S6 Replication of WTCCC associations in GAIN+TGEN as a function of power. Plot of the power to detect an effect in GAIN+TGEN based on odds ratio and minor allele frequency reported in WTCCC. Each point represents a SNP and is plotted according to OR (rounded to 2 digits) in WTCCC and power to detect an effect in the GAIN+TGEN sample at alpha = 0.05. The points are color coded to MAF in WTCCC (rounded to 2 digits). Text on the left hand side indicates the number of SNPs within each power decile that were associated at P<0.05 in GAIN+TGEN. Thus, of the 1,979 SNPs with power between 0.8 and 0.9, 165 (8.3%) were associated at P<0.05.
(JPG)

Figure S7 Power*near exon interaction significance for varying distance from exons. SNPs were categorized by distance from any exon (RefSeq). For each distance cut-off, whether a SNP was near an exon was used as a predictor of replication in the test dataset. The −log10P value is shown for the power*near_exon term in the logistic regression test.
(PDF)

Figure S8 Enrichment of replication at P<0.05 in the WTCCC based on power calculated from GAIN+TGEN study. For different classes of SNPs, the smoothed spline is shown for the proportion of SNPs showing association at P<0.05 in the WTCCC-BD dataset as a function of power based on the GAIN+TGEN dataset.
(PDF)

Table S1 Bipolar Disorder Case Characteristics.
(XLS)

Table S2 Top hits for GAIN+TGEN GWAS. *top SNP was imputed. #SNPs @ P<10^−5^ indicates the number of SNPs within 150 kb on either side of the top SNP that were associated at P<10^−5^ and is reported as genotyped/imputed. SNPs are reported in genome forward (b36) orientation.
(XLS)

Table S3 Association results for SNPs implicated in previous studies. *results are from imputed data.
(XLS)

Table S4 Top hits for GAIN+TGEN and WTCCC meta-analysis. SNPs are reported in genome forward (b36) orientation. Odds ratios are reported for A1 allele. P_Het_ = Heterogeneity P-value from Cochrane's Q statistic.
(XLS)

Table S5 P-values showing that having the same direction of effect is predictive of replication when the cases have the same phenotype (bipolar disorder). P-values for logistic regression from two models M1 and M2. M1: Replication in GAIN+TGEN∼ effect size in same direction in WTCCC and GAIN+TGEN. M2: Replication in GAIN+TGEN∼powerWTCCC+effect size in same direction in WTCCC and GAIN+TGEN+powerWTCCC* effect size in same direction. Header abbreviations: BD: bipolar disorder, CD: Crohn's disease, CAD: coronary artery disease, HT: hypertension, RA: rheumatoid arthritis, T1D: type 1 diabetes, T2D: type 2 diabetes.
(XLS)
