# Supplementary figures and images for: Genome-Wide Association of Bipolar Disorder Suggests an Enrichment of Replicable Associations in Regions near Genes
Source: PLoS Genet. 2011 Jun 30;7(6):e1002134. doi: 10.1371/journal.pgen.1002134 (PMC3128104; doi:10.1371/journal.pgen.1002134)

# A. GAIN-TGEN

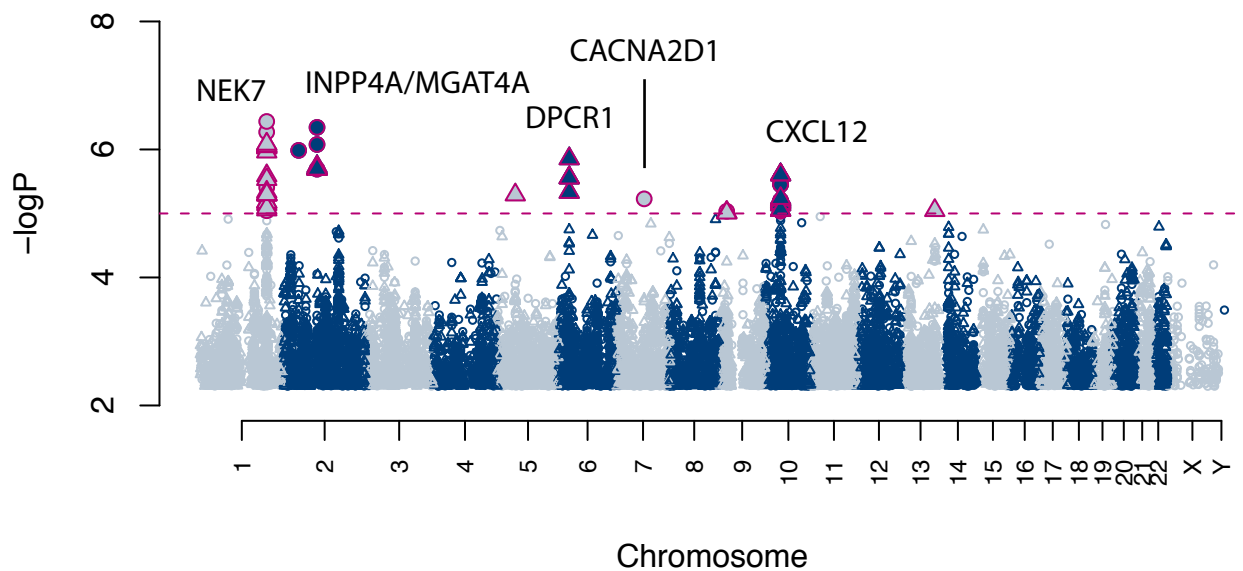

# B. GAIN-TGEN + WTCCC Meta-analysis

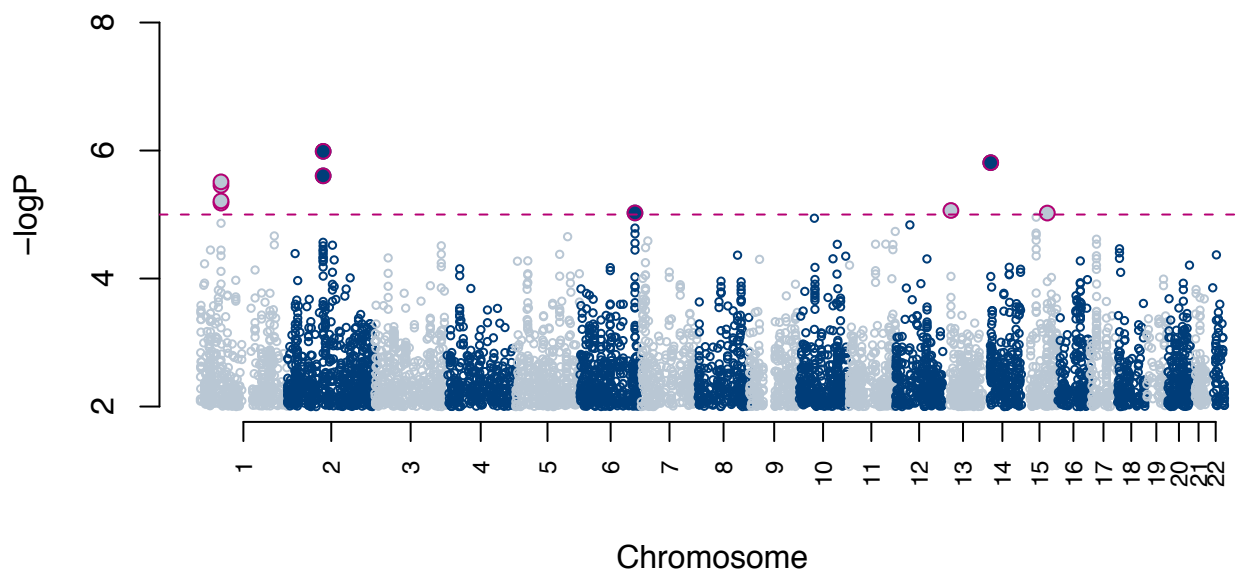

Supplement: Figure S1 — Manhattan plots of GAIN+TGEN and Meta-analysis −logP values. Manhattan plots for A) GAIN+TGEN and B) GAIN+TGEN+WTCCC BP meta-analysis. Points are indicated as circles for genotyped data and triangles for imputed data. Points are larger and circled in pink if the P-value<10−5, also indicated by a pink dotted line. (PDF) [file pgen.1002134.s001.pdf]

**rs1927252**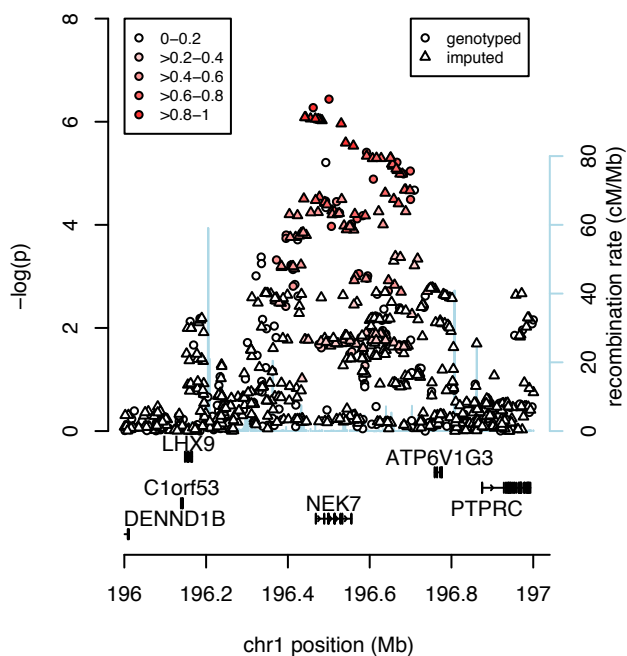**rs17498753**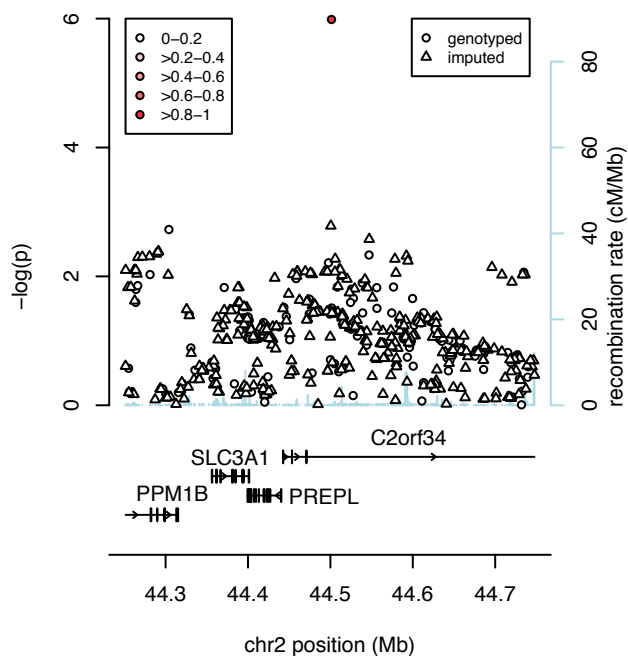**rs7560357**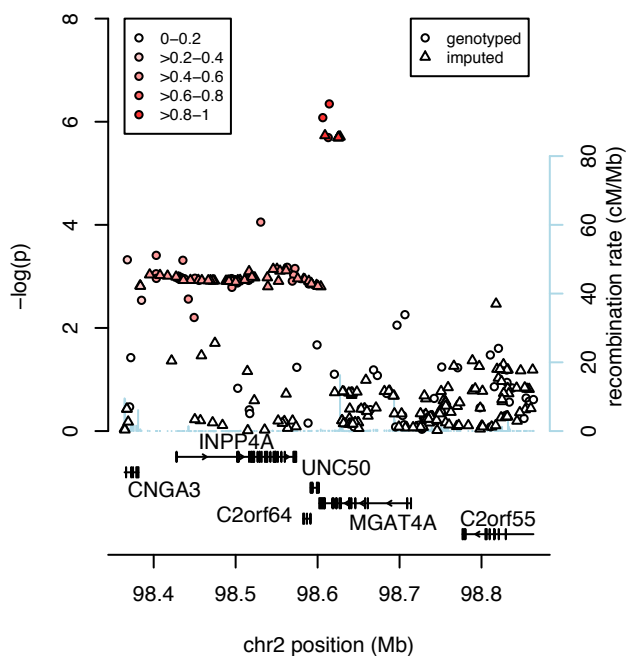**rs7703177**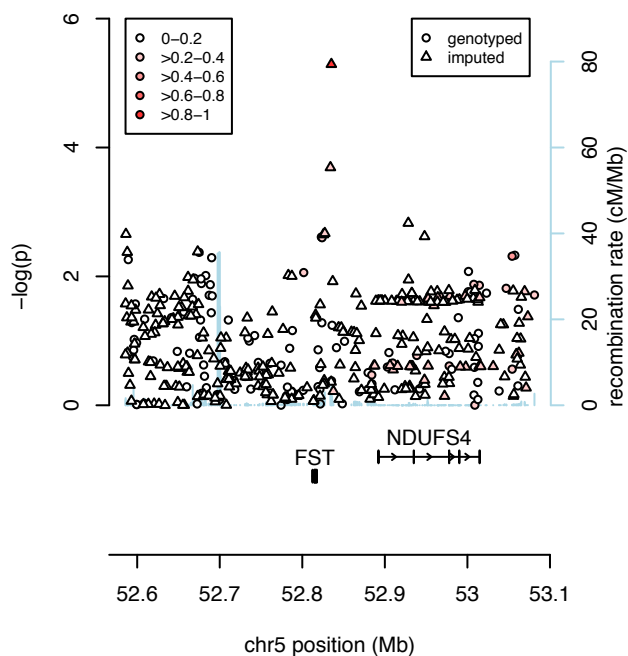

rs7755802

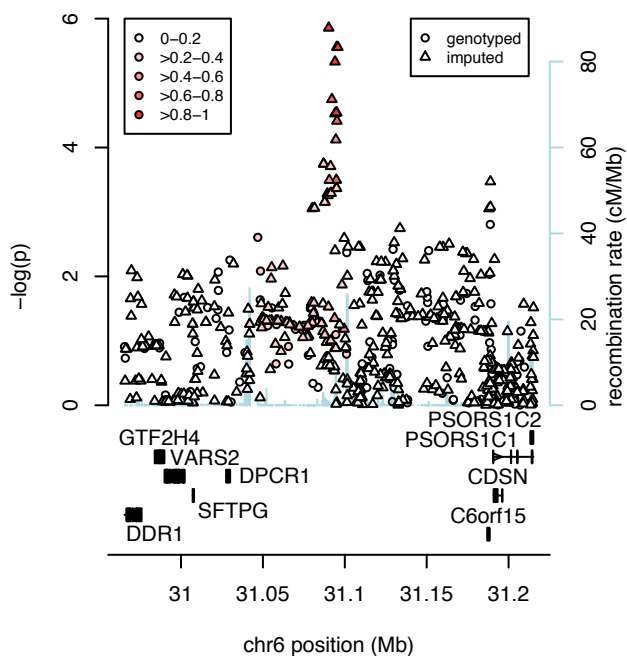

rs2367911

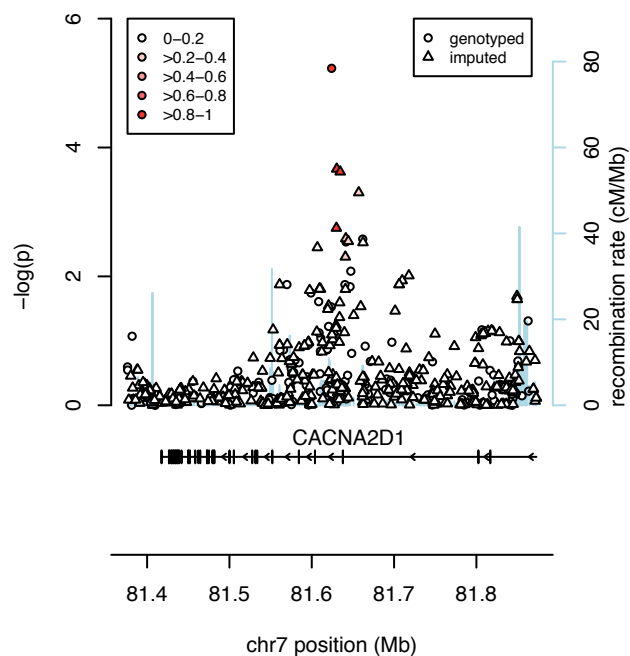

rs6475738

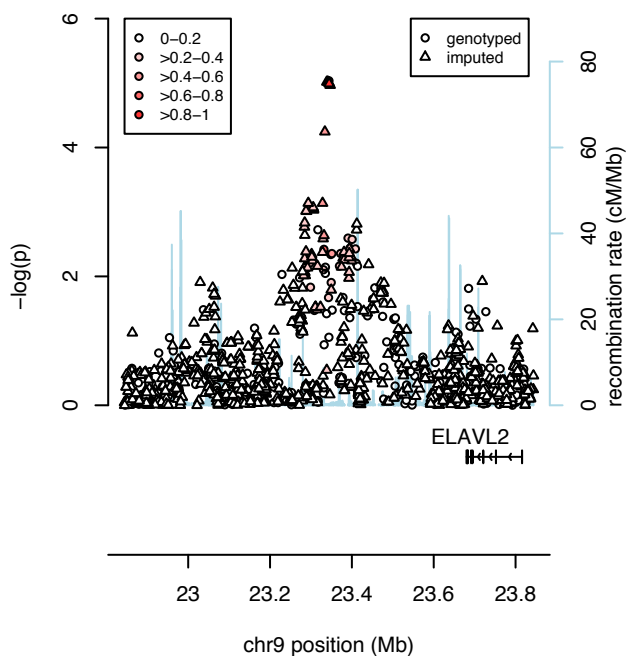

rs1585913

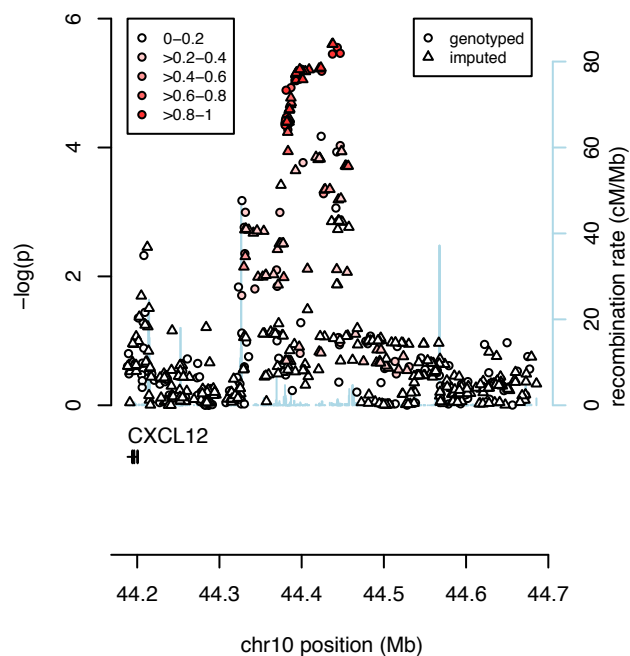

**rs293969**

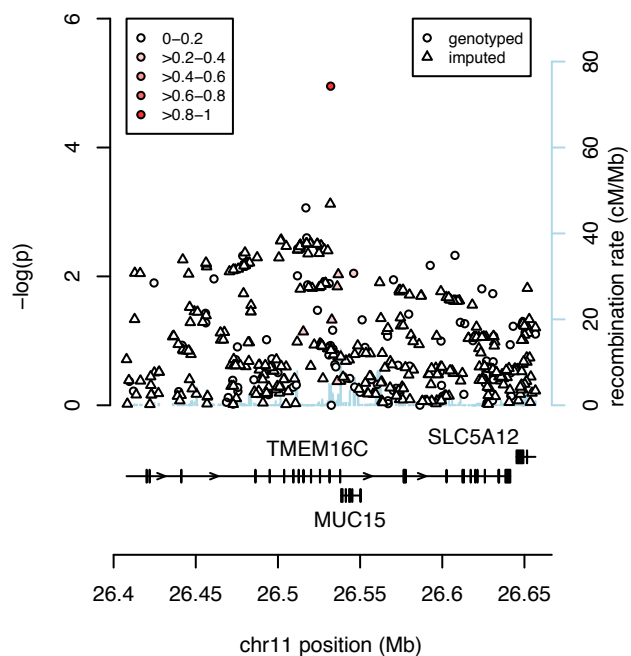

**rs9554603**

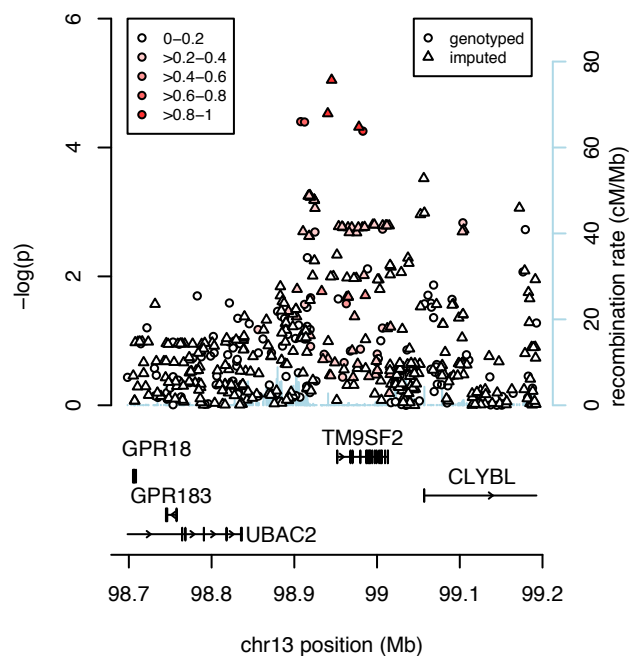

Supplement: Figure S2 — Regional plots for GAIN+TGEN GWAS. Associations reaching P<10−5 in the GAIN+TGEN GWAS are shown with nearby markers. The X-axis indicates the chromosomal position based on NCBI build 36. Genes are shown based on their locations in RefSeq. Arrows indicate the direction of transcription. Points are color coded according to LD within the GAIN+TGEN study with the most associated SNP. For imputed markers, LD was calculated as correlation in PLINK using best estimates for genotypes. Recombination rate is indicated on the second y-axis and color-coded in light blue. Cluster plots for singleton SNPs such as rs17498753 and rs293969 were inspected and found to be of good quality. (PDF) [file pgen.1002134.s002.pdf]

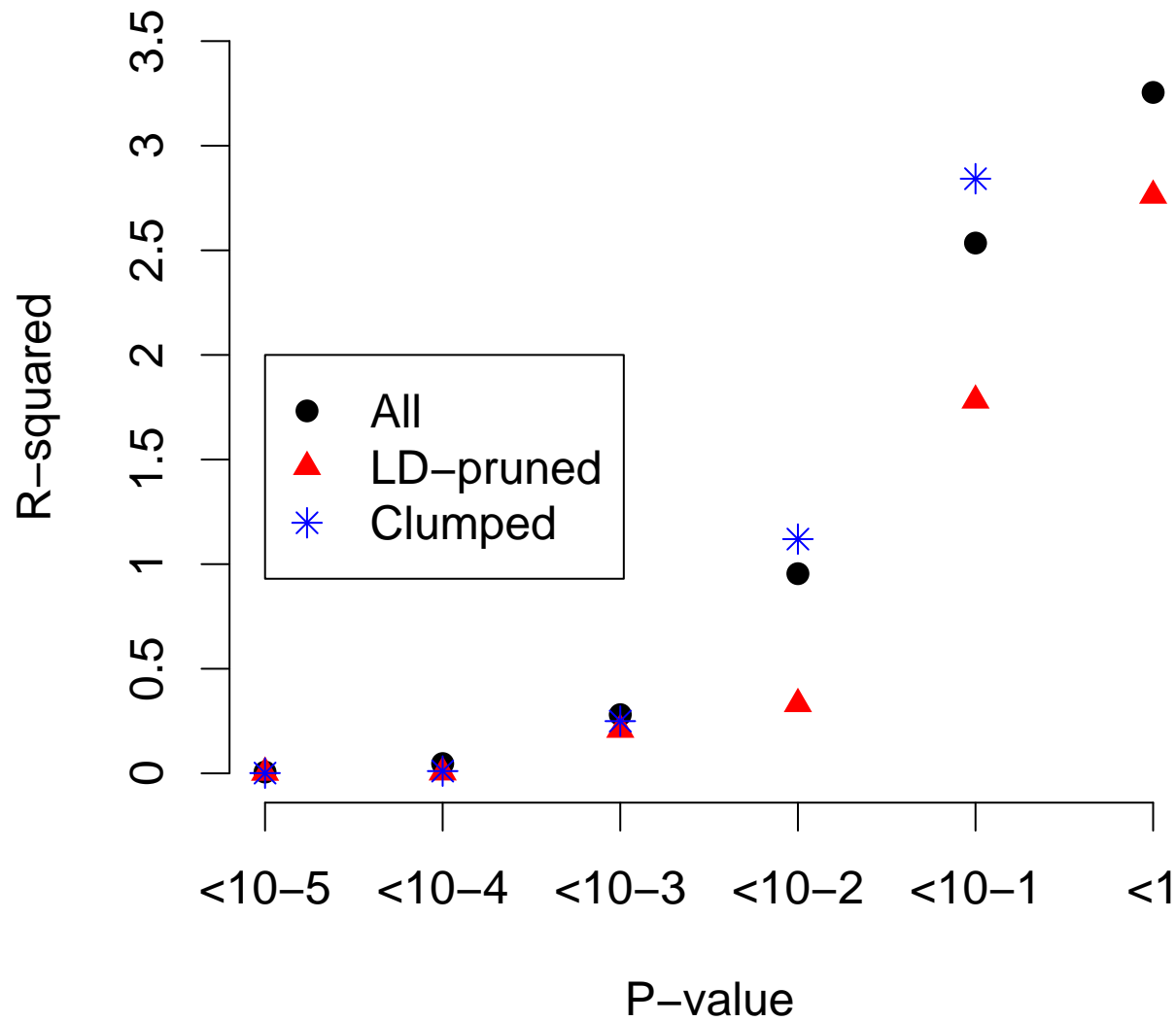

Supplement: Figure S3 — Variance in diagnosis explained in GAIN+TGEN by score summed over using subsets of SNPs from the WTCCC-BD study. A score was calculated for each individual based on their genotype and the odds ratio of each SNP from the WTCCC-BD study. The score was used to predict case-control status in GAIN+TGEN and shown are the pseudo-R2 values from logistic regression for subsets of SNPs used to calculate a score. SNPs were grouped by P-value, with each category adding progressively more SNPs with weaker P-values until all SNPs are included. LD-pruned SNPs were pruned to be in linkage equilibrium (r2<0.5). Clumped SNPs were pruned to index SNPs to ensure independent associations. (PDF) [file pgen.1002134.s003.pdf]

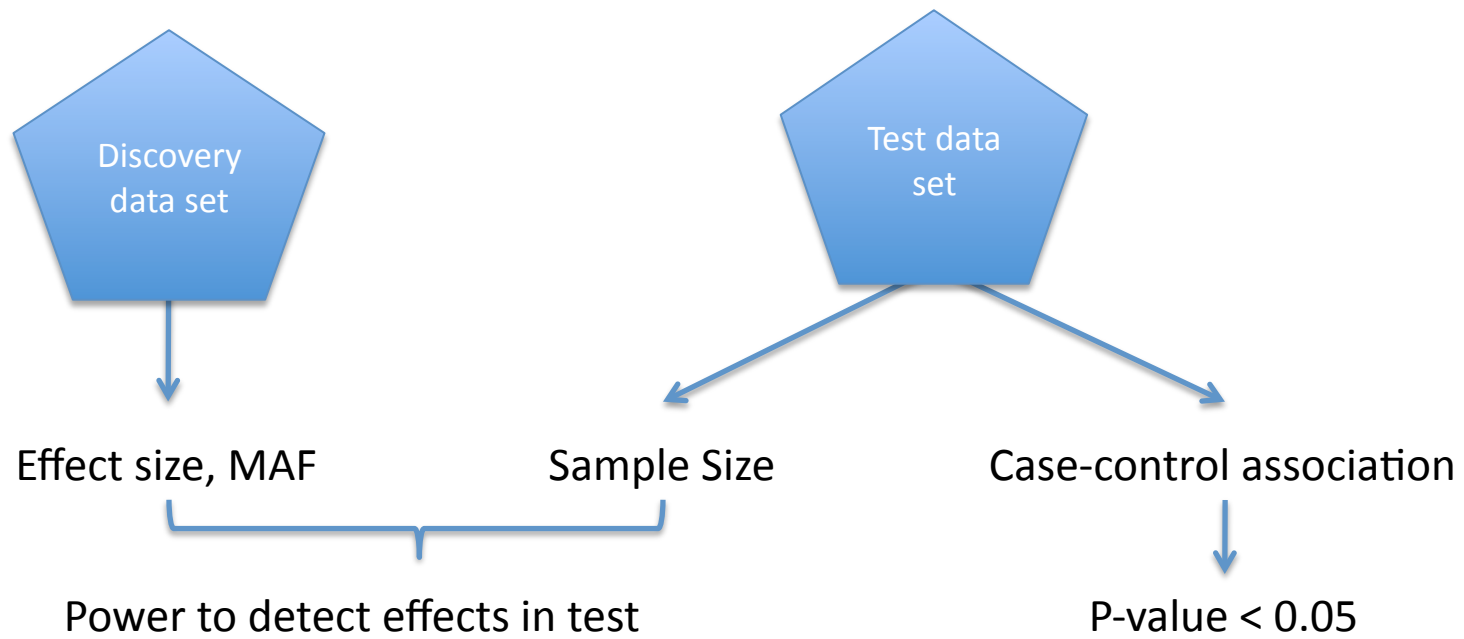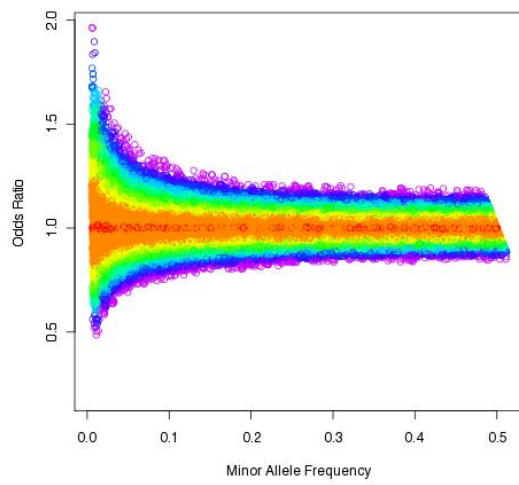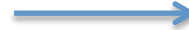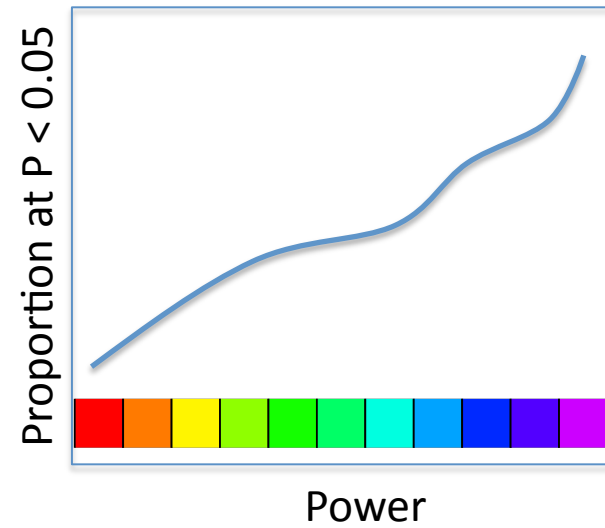

Supplement: Figure S4 — Schematic of test for replication as a function of power. (PDF) [file pgen.1002134.s004.pdf]

## A. Bipolar disorder

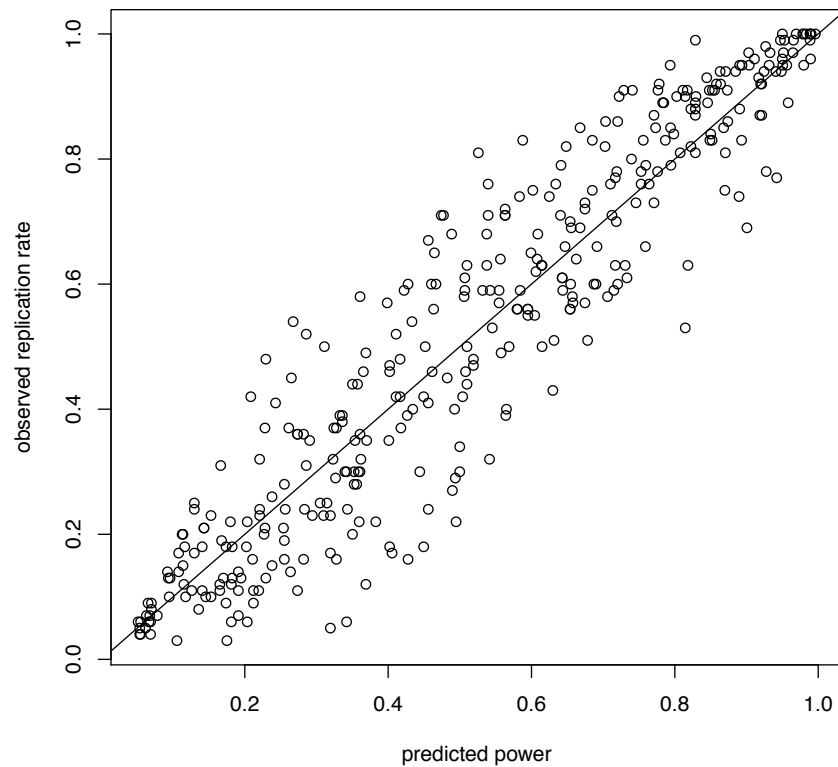

## B. Height

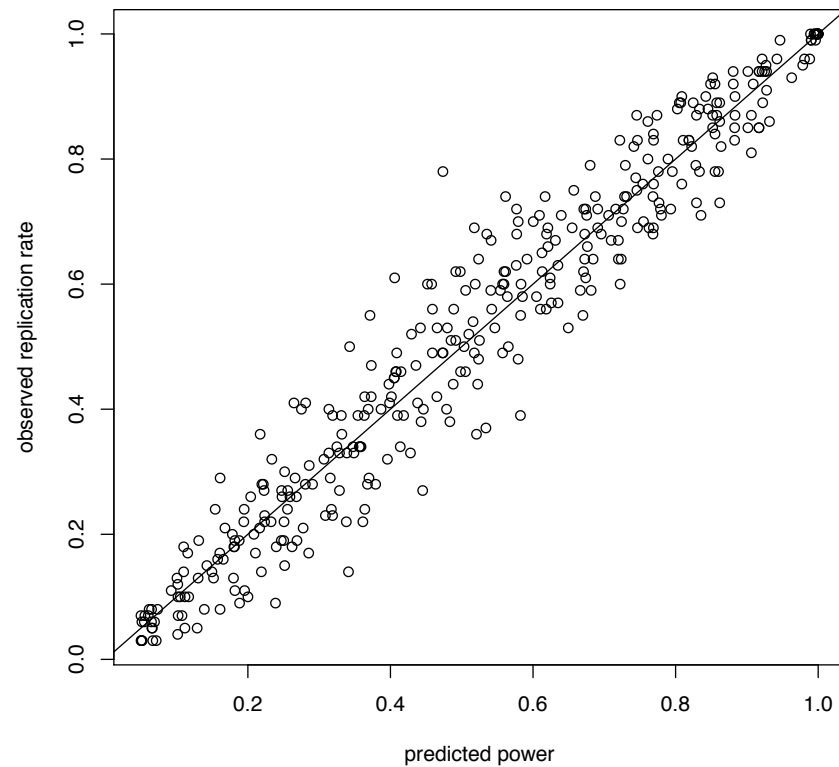

Supplement: Figure S5 — Simulation studies show a linear relationship between power and replication. A sample of SNPs were selected from A) BD or B) height and the effects were simulated in a population of 1,000,000 individuals assuming a baseline prevalence of 1% for BD and a normal distribution for height. Random case-control (BD) or population-based (height) samples were taken such that they matched the observed sample sizes. For each SNP, 100 of these samples were taken and the observed replication rate corresponds to the proportion of samples where the P-value was less than 0.05. A line is drawn at y = x. (PDF) [file pgen.1002134.s005.pdf]

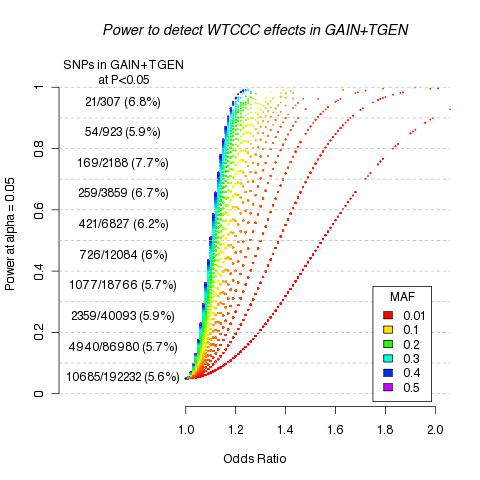

Supplement: Figure S6 — Replication of WTCCC associations in GAIN+TGEN as a function of power. Plot of the power to detect an effect in GAIN+TGEN based on odds ratio and minor allele frequency reported in WTCCC. Each point represents a SNP and is plotted according to OR (rounded to 2 digits) in WTCCC and power to detect an effect in the GAIN+TGEN sample at alpha = 0.05. The points are color coded to MAF in WTCCC (rounded to 2 digits). Text on the left hand side indicates the number of SNPs within each power decile that were associated at P<0.05 in GAIN+TGEN. Thus, of the 1,979 SNPs with power between 0.8 and 0.9, 165 (8.3%) were associated at P<0.05. (JPG) [file pgen.1002134.s006.jpg]

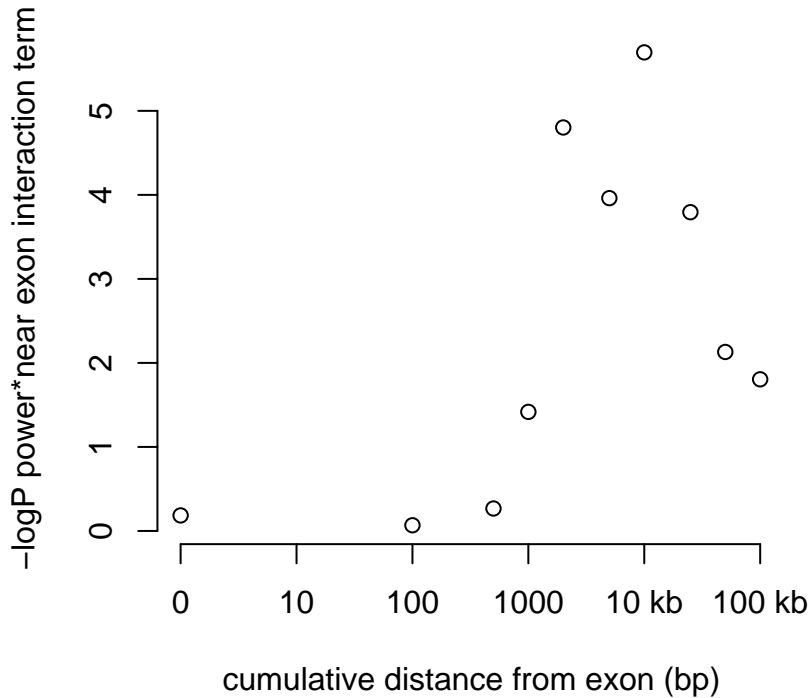

Supplement: Figure S7 — Power*near exon interaction significance for varying distance from exons. SNPs were categorized by distance from any exon (RefSeq). For each distance cut-off, whether a SNP was near an exon was used as a predictor of replication in the test dataset. The −log10P value is shown for the power*near_exon term in the logistic regression test. (PDF) [file pgen.1002134.s007.pdf]

proportion of SNPs @  $P < 0.05$  in WTCCC

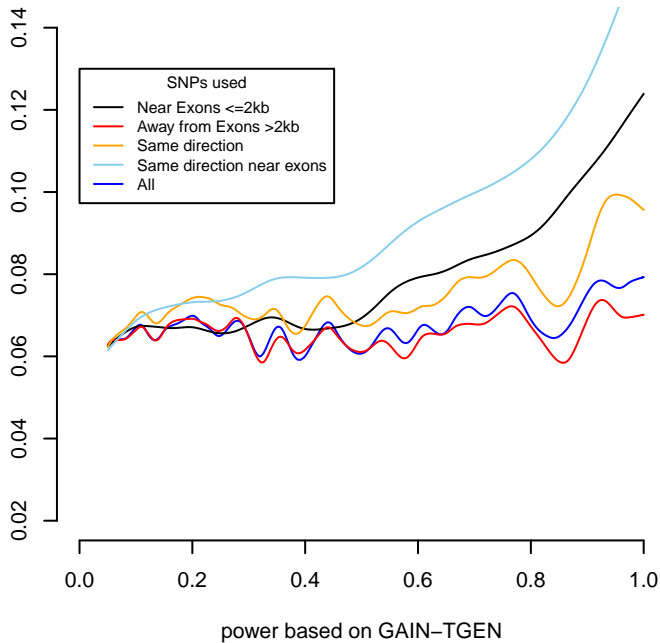

Supplement: Figure S8 — Enrichment of replication at P<0.05 in the WTCCC based on power calculated from GAIN+TGEN study. For different classes of SNPs, the smoothed spline is shown for the proportion of SNPs showing association at P<0.05 in the WTCCC-BD dataset as a function of power based on the GAIN+TGEN dataset. (PDF) [file pgen.1002134.s008.pdf]
